# Case report: Muscle involvement in a Chinese patient with *TRNT1*-related disorder

**DOI:** 10.3389/fped.2023.1160107

**Published:** 2023-05-05

**Authors:** Cui-Jie Wei, Yi-Dan Liu, Yan-Ling Yang, Yuan Wu, Jie-Yu Liu, Xing-Zhi Chang, Ying Hua, Yu-He Liu, Hui Xiong

**Affiliations:** ^1^ Department of Pediatrics, Peking University First Hospital, Beijing, China; ^2^ Department of Otolaryngology Head and Neck Surgery, Capital Medical University Affiliated Beijing Friendship Hospital, Beijing, China

**Keywords:** TRNT1, mitochondrial myopathy, hyperCKemia, sensorineural hearing loss, sideroblastic anemia, developmental delay

## Abstract

The *TRNT1* gene encodes tRNA nucleotidyltransferase 1, which catalyzes the addition of cytosine-cytosine-adenosine (CCA) to the ends of cytoplasmic and mitochondrial tRNAs. The most common clinical phenotype associated with *TRNT1* is autosomal recessive sideroblastic anemia with B-cell immunodeficiency, periodic fever, and developmental delay (SIFD). Muscle involvement has rarely been reported in *TRNT1*-related disorders. Here we report a Chinese patient with incomplete SIFD and hyperCKemia, and explored the skeletal muscle pathological changes. The patient was a 3-year-old boy with sensorineural hearing loss, sideroblastic anemia, and developmental delay since infancy. At the age of 11 months, significantly increased levels of creatine kinase were noted, accompanied by mild muscle weakness. Whole-exome sequencing revealed compound heterozygous variants of the *TRNT1* gene, c.443C > T (p.Ala148Val) and c.692C > G (p.Ala231Gly), in the patient. Western blot showed a decreased expression of TRNT1 and cytochrome c oxidase subunit IV (COX IV) in the skeletal muscle of the patient. Electron microscopy observation of skeletal muscle pathology revealed abnormal mitochondria of various sizes and shapes, supporting a diagnosis of mitochondrial myopathy. The present case indicates that in addition to the classic SIFD phenotype, *TRNT1* mutations can cause mitochondrial myopathy, a rare clinical phenotype of *TRNT1*-related disorders.

## Introduction

The *TRNT1* gene encodes tRNA nucleotidyltransferase 1, which is widely present in tissues in the whole body. The enzyme catalyzes the addition of cytosine-cytosine-adenosine (CCA) to the 3-prime end of tRNA precursors both in the cytoplasm and mitochondria (1–3). Clinical phenotypes associated with *TRNT1* include autosomal recessive sideroblastic anemia with B-cell immunodeficiency, periodic fever, and developmental delay (SIFD, OMIM: 616084), and retinitis pigmentosa with erythrocytic microcytosis (OMIM: 616959). Till now, a total of 58 patients with SIFD and 41 mutations have been reported (4). SIFD is a autoinflammatory multisystem disorder, and the phenotypic spectrum is still emerging, with some specific common features but a diverse set of clinical phenotypes and patterns of system involvement described, thus it would be more appropriate to consider this syndrome as a *TRNT1*-related disorder (4). Muscle involvement has rarely been reported in *TRNT1*-related disorders. Only one patient has been reported to have a mild elevation of serum creatine kinase (CK) up to 383 U/L (5). Three other patients were found to have a decreased enzyme activity of the muscle mitochondrial respiratory chain, but none of them had clinical symptoms, such as muscle weakness or hyperCKemia (3, 5).

In this study, we report a Chinese child with *TRNT1* gene mutations who had an incomplete SIFD phenotype and significantly elevated levels of CK. A skeletal muscle pathology examination was performed to explore the mechanism of muscle involvement.

## Case description

The index case of this study was a 3-year-old boy who was admitted to the hospital due to sensorineural hearing loss, sideroblastic anemia, and developmental delay. He was born to non-consanguineous parents and was conceived by *in vitro* fertilization. The birth was uneventful at 37 ^+ 3^ weeks of gestation. He failed hearing screening after birth and was diagnosed with severe all-frequency sensorineural hearing loss at the age of 6 months, based on behavioral audiometry, auditory brainstem response (ABR), and distortion product otoacoustic emissions (DPOAE). After being fitted with a hearing aid, he responded to loud sounds. At the age of 8 months, routine examination showed mild microcytic hypochromic anemia with minimum hemoglobin of 88 g/L. Serum iron, ferritin, and serum transferrin saturation levels were normal. Iron staining of the bone marrow showed that ring sideroblasts accounted for 5%, supporting a diagnosis of sideroblastic anemia. During follow-up, the patient's hemoglobin level was 100–110 g/L. At the age of 11 months, hyperCKemia was noted in a routine examination. During follow-up, CK remained elevated, with a minimum of 724 U/L and a maximum of 13,280 U/L (concurrent serum myoglobin 1,106 ng/ml, reference value 0–70). Most of the time, the CK level fluctuated between 3,000–8,000 U/L (reference value 25–195). There was no relationship between CK value and exercise or infection. The patient was always weak and easily tired but had never experienced dark-colored urine. Psychomotor and physical development was abnormally delayed without seizures. He could sit unaided at 6 months and walk with support at 12 months but was never able to walk independently. He made little eye contact with others and still could not understand instructions or say any words. His growth also slowed down after the age of 1 year. His current weight and height of 12.7 kg and 90 cm, respectively, were less than the 3rd percentile of the same age and sex.

On admission, physical examination showed that the muscle strength was reduced in all limbs to grade 4 (based on the MRC scale), accompanied by significant hypotonia. Knee tendon and Achilles tendon reflexes were symmetrically elicited. After admission, laboratory examination revealed hemoglobin was 112 g/L. The immune function test showed that immunoglobulin G was reduced to 2.93 g/L (reference value 7–17), while peripheral blood T and B lymphocyte subsets were within the normal range. He had hyperlactatemia (3.0 mmol/L). The CK level was 3,321 U/L, while the serum myoglobin result was 330 ng/ml. Echocardiography showed ventricular septum hypertrophy. Visual evoked response and ABR testing could not elicit any waveform. Brain magnetic resonance imaging showed bilateral cerebral white matter hypomyelination. The Griffiths Mental Development Scale-Chinese was used to assess the development and showed a marked delay in the “Locomotor” (equivalent age of 11 months), “Personal-social” (equivalent age of 6.5 months), “Hearing and Language” (equivalent age of 5.5 months), “Hand-eye coordination” (equivalent age of 7.5 months) and “Performance” (equivalent age of 6 months) areas.

**Figure 1:**
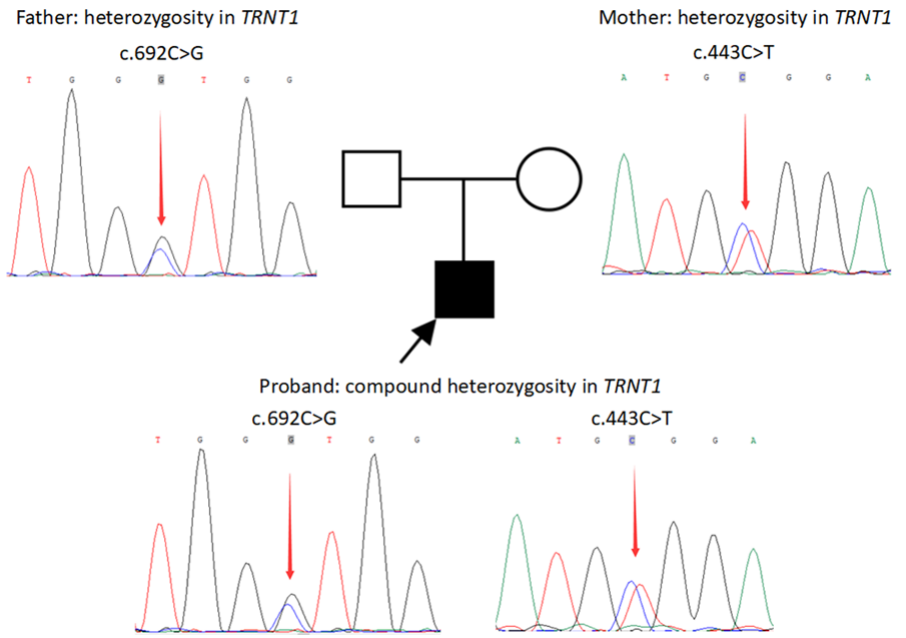
Variants in the TRNT1 gene in the family revealed by sanger sequencing. Compound heterozygous variants in *TRNT1* (NM_182916.2) of the patient: c.443C > T derived from the mother and c.692C > G derived from the father.

Trio-based whole-exome sequencing (Trio-WES) was performed (See the Supplementary Material for details). Candidate variants were validated by Sanger sequencing. Compound heterozygous variants were identified in *TRNT1* (NM_182916.2), with c.443C > T derived from the mother and c.692C > G from the father (Figure 1). The pathogenicity of c.443C > T had been previously proved in a patient with SIFD (3). The variant c.692C > G was a novel variant and it was not present in the gnomAD and gnomAD_EAS databases. According to the American College of Medical Genetics and Genomics guidelines (6), the two variants could be considered “pathogenic” and “likely pathogenic”, respectively.

**Figure 2:**
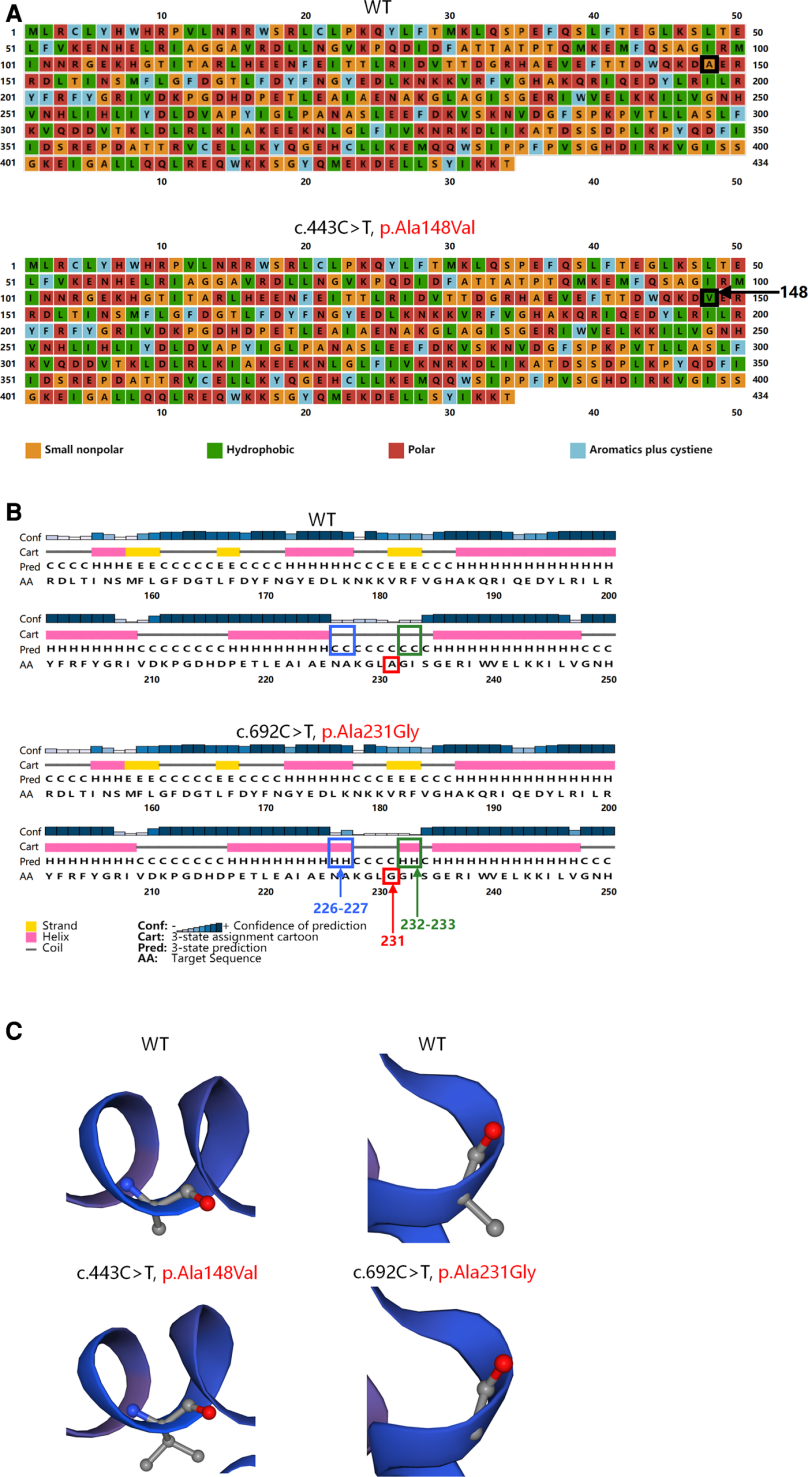
Structures of the wild-type and mutated TRNT1 proteins. (**A**) Variant c.443C > T (p.Ala148Val) led to a substitution of hydrophobic valine (green) for small nonpolar alanine (orange) at position 148 (black arrow) showed by PSIPRED. (**B**) Variant c.692C > G (p.Ala231Gly) (red arrow) resulted in changes in secondary structures of the adjacent amino acids 226–227 (blue arrow) and 232–233 (green arrow) from a coil to a helix predicted by PSIPRED. (**C**) Variant c.443C > T (p.Ala148Val) and variant c.692C > G (p.Ala231Gly) caused changes in local spatial structure of TRNT1 proteins predicted by SWISS-MODEL.

PSIPRED V4.0 (http://bioinf.cs.ucl.ac.uk/psipred/) was further used to predict the secondary structures of the mutated and wild-type TRNT1 proteins. The variant c.443C > T (p.Ala148Val) was a missense variant that led to a change in the polarity of amino acids (Figure 2A). The other missense variant c.692C > G (p.Ala231Gly) resulted in changes in the secondary structures of the adjacent amino acids 226–227 and 232–233 from a coil to a helix (Figure 2B). The changes in local spatial structure were predicted by SWISS-MODEL (https://swissmodel.expasy.org/) (Figure 2C). A single nucleotide polymorphism (SNP) array, mitochondrial DNA sequencing, and trio-based whole-genome sequencing (trio-WGS) that were performed revealed no other variants associated with the phenotype of the patient (See the Supplementary Material for details).

**Figure 3:**
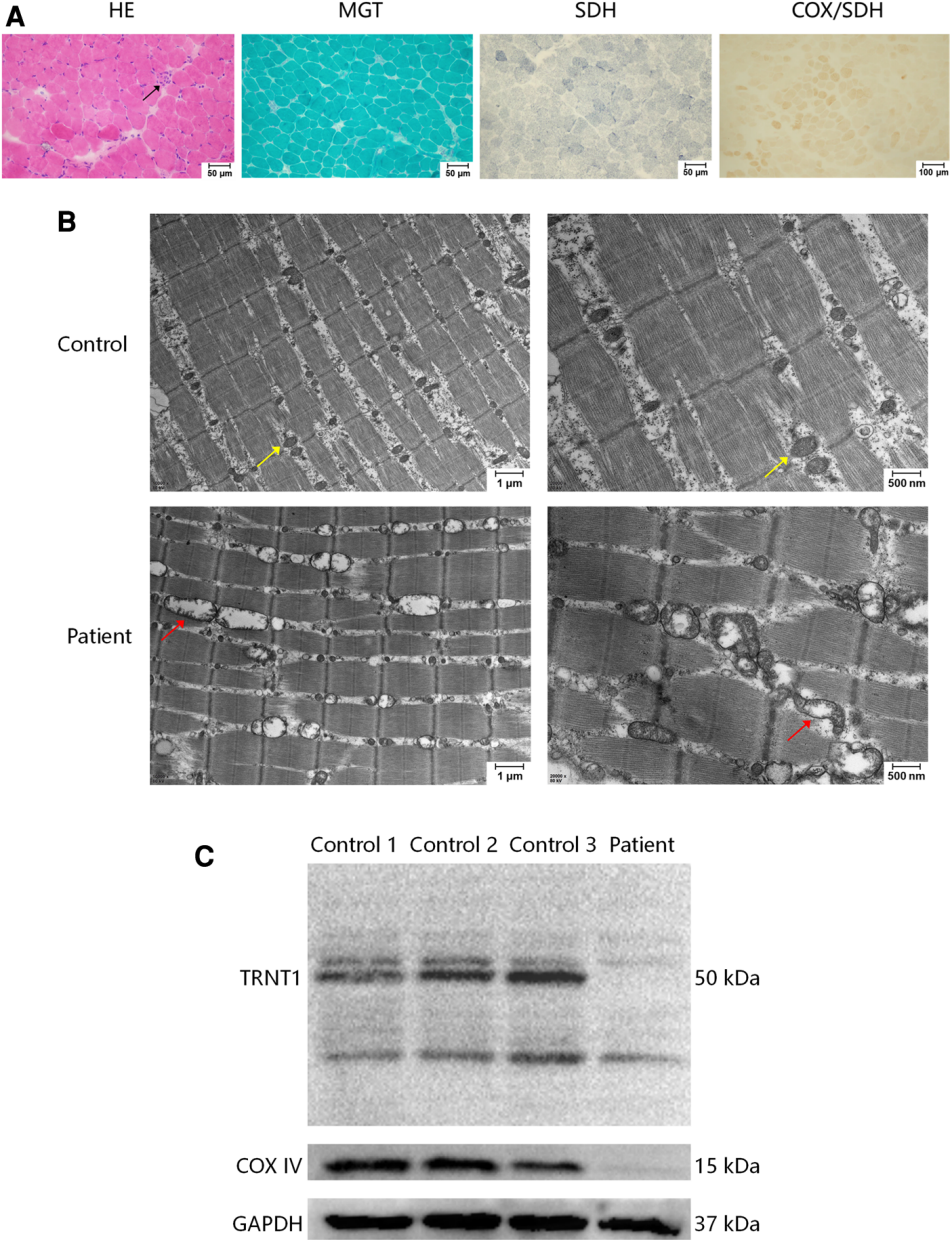
Muscle biopsy of right quadriceps muscle. (**A**) In a light microscopy, haematoxylin and eosin (HE) stain showed that polygonal muscle fibers were closely packed to each other, with occasional necrosis muscle fibers (arrow). No abnormities were detected in modified Gomori trichrome (MGT) staining, succinate dehydrogenase (SDH) staining, and combined cytochrome oxidase (COX)/SDH staining. (**B**) Electron microscopy showed that the mitochondria around the muscle fibers were of different sizes and shapes. Some were empty or had discontinued ridges (red arrows). The control showed normal mitochondria (yellow arrows). (**C**) Western blot of TRNT1, cytochrome c oxidase subunit IV (COX IV), and glyceraldehyde-3-phosphate dehydrogenase (GAPDH) in human skeletal muscle samples of 3 independent controls and the patient. The patient showed a decreased expression of TRNT1 and COX IV. The primary antibodies are anti-TRNT1 polyclonal antibody (Abcam, ab224536), anti-COX IV monoclonal antibody (Gene-Protein Link, P01L08), and anti-GAPDH monoclonal antibody (Cell Signaling Technology, #2118).

To clarify the cause of the high CK level, the right quadriceps muscle was biopsied. Light microscopy revealed slight pathological changes. Hematoxylin and eosin (HE) staining showed occasional necrotic muscle fibers. No typical “ragged-red” fibers (RRFs), “ragged-blue” fibers (RBFs), or cytochrome c oxidase (COX)-negative muscle fibers were found (Figure 3A). Electron microscopy showed that the mitochondria around the muscle fibers had different sizes and shapes. Some were empty or had discontinued ridges, supporting a diagnosis of mitochondrial myopathy (Figure 3B). Western blot showed a significantly decreased expression of TRNT1 in the skeletal muscle of the patient. A decreased expression of cytochrome c oxidase subunit IV (COX IV) protein was also detected (Figure 3C), indicating a damage of muscle mitochondrial respiratory chain complex IV (See the Supplementary Material for details of Western blot).

Above all, according to Bernier et al. (2002) criteria for the diagnosis of mitochondrial respiratory chain disorders (7), the patient met the major clinical criteria for unexplained multisystem involvement, and other diseases were excluded by metabolic screening and genetic testing. He also met three minor diagnostic criteria: widespread electron microscopic mitochondrial abnormality in histology, a nuclear mutation of probable pathogenicity, and identification of hyperlactatemia in metabolism. Therefore, a definite diagnosis of mitochondrial disorders was established.

## Discussion

TRNT1 is an essential enzyme that catalyzes the addition of the CCA trinucleotides terminus to the 3-prime end of tRNA precursors, which is necessary for aminoacylation (8). Mutations in the *TRNT1* gene lead to an abnormal modification of cytoplasmic and mitochondrial tRNA, which further affects the formation of the mitochondrial respiratory chain complex, resulting in an oxidative phosphorylation defect and insufficient ATP supply. SIFD is the classic phenotype caused by *TRNT1* gene mutations, which mostly involve the immune, blood, and nervous systems, but can also affect vision, hearing, heart, and kidneys (5, 9–12). However, only 65% of patients showed the complete phenotype characterized by anemia, recurrent fever, immunodeficiency and neurodevelopmental delay (4). In the present case, the child had an incomplete SIFD phenotype featuring low IgG, sideroblastic anemia, and developmental delay, but lacked the characteristic manifestation of periodic fever and B-cell immunodeficiency. Notably, this patient was accompanied by a significantly elevated CK, which has not been reported in the previous literature.

The human TRNT1 protein contains 434 amino acids and has two domains: a polymerase head domain (amino acids 59–182) and a probable RNA and SrmB-binding site of polymerase A (amino acids 215–271) (http://pfam.xfam.org/protein/Q96Q11). The variants c.443C > T (p.Ala148Val) and 692C > G (p.Ala231Gly) are located in the above two functional domains, respectively. And both variants are predicted to affect the spatial structure of the TRNT1 protein. Additionally, a decreased expression of TRNT1 protein has been observed in the skeletal muscle of the patient. SNP array, mitochondrial DNA sequencing, and trio-WGS found no other mutations that could explain myopathy. Therefore, we speculate that the hyperCKemia in this patient was likely attributed to *TRNT1* mutations.

Skeletal muscles, as high energy-consuming tissues, are often involved in mitochondrial disease, and the associated clinical manifestations range from asymptomatic hyperCKemia, varying degrees of muscle weakness, to rhabdomyolysis induced by infection or exercise. However, there are few reports of muscle involvement in *TRNT1*-related disorders. Only one patient has been reported to have a mild elevation of CK to 383 U/L (5). Three other patients were found to have a decreased enzyme activity of muscle mitochondrial respiratory chain, but none of them had symptoms of myopathy, such as muscle weakness or hyperCKemia (3, 5). Unlike previous literature, the patients in this study presented with markedly elevated CK and mild muscle weakness. Moreover, his CK fluctuated greatly, with a maximum of 13,280 U/L, which was also in line with the characteristics of mitochondrial myopathy. The muscle pathology on light microscopy was relatively mild, and no RRFs or COX-negative muscle fibers were found. We speculate the reasons for the lack of RRFs or COX-negative muscle fibers: the first reason is that RRFs are rarely seen in patients with mitochondrial myopathy under 5 years of age; the second reason is that COX-negative muscle fibers are more common in mitochondrial gene mutations, but relatively rare for nuclear gene mutations. Electron microscopy observation of skeletal muscle pathology revealed abnormal mitochondria of various sizes and shapes, supporting a diagnosis of mitochondrial myopathy. Sasarman F et al. reported a patient harboring homozygous c.443C > T mutation in *TRNT1* whose muscle autopsy showed enlarged mitochondria under electron microscopy (3), similar to our patient. Therefore, electron microscopy may be more helpful for discovering mitochondrial abnormalities in muscles in *TRNT1*-related disorders. Unfortunately, due to the limited amount of muscle samples of the patient, the enzyme activity of the muscle mitochondrial respiratory chain was not performed. But COX IV deficiency in the patient was detected by western blot, hinting a damage of muscle mitochondrial respiratory chain complex IV, whose activity was reduced in two previous reported patients with *TRNT1* mutations (3, 5). Based on muscle weakness, significantly increased levels of CK, and the effect of *TRNT1* mutations on muscle mitochondria, we diagnosed the patient with mitochondrial myopathy and gave him a “cocktail” medicine including levocarnitine, coenzyme Q10, and so on. After the treatment, his motor ability improved gradually, without developmental regression after infection.

In conclusion, this study describes a case with incomplete SIFD with prominent hyperCKemia caused by compound heterozygous variants in the *TRNT1* gene, which indicates that in addition to the classic SIFD phenotype, *TRNT1* mutations can cause mitochondrial myopathy.

## Data Availability

The datasets presented in this article are not readily available because of ethical and privacy restrictions. Requests to access the datasets should be directed to the corresponding author/s.
