# Supplementary figures and images for: Case report: Muscle involvement in a Chinese patient with TRNT1-related disorder
Source: Front Pediatr. 2023 May 5;11:1160107. doi: 10.3389/fped.2023.1160107 (PMC10196124; doi:10.3389/fped.2023.1160107)

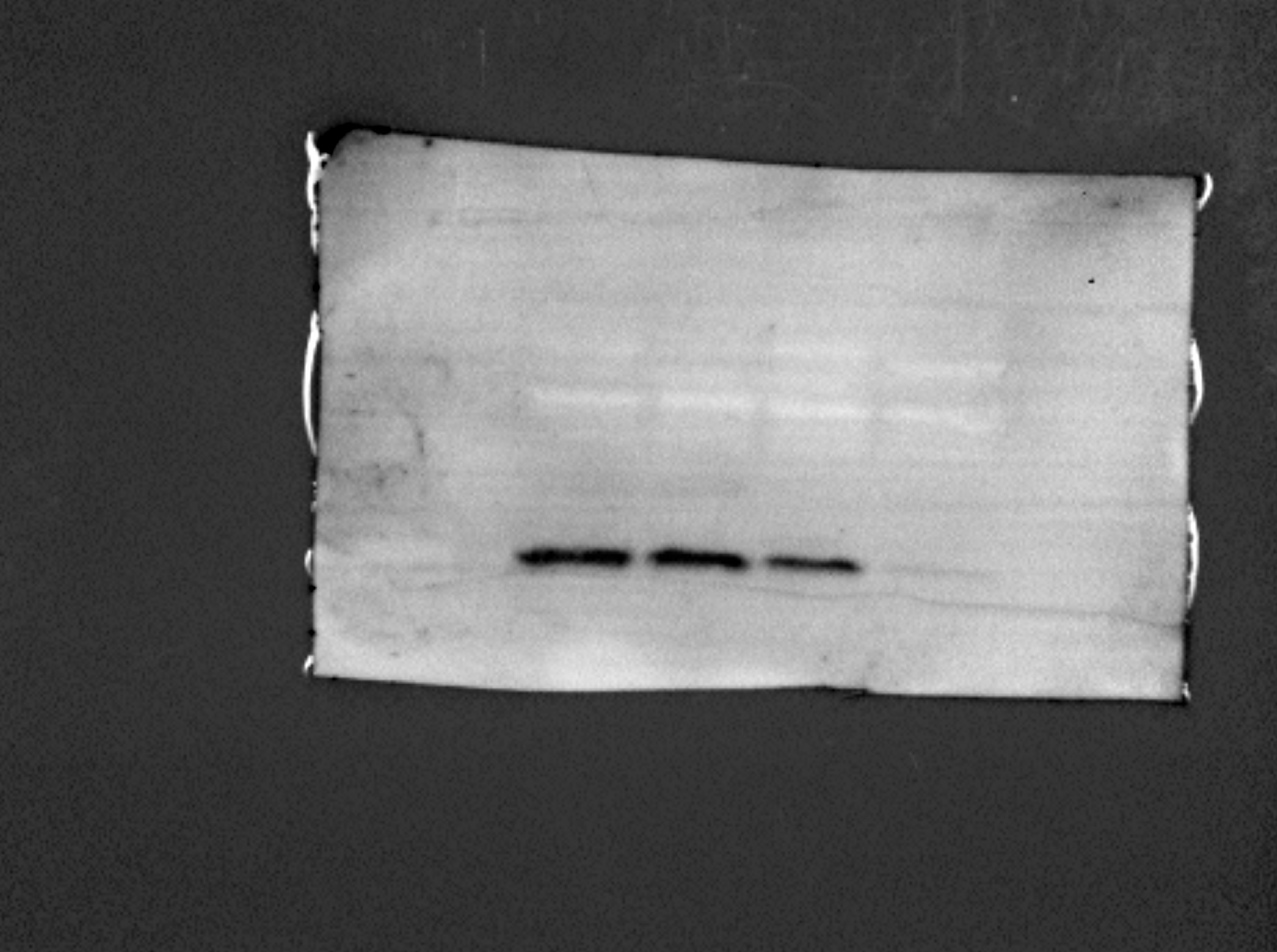

Supplement: Supplementary file 1 [file Datasheet1.zip › Data Sheet 1_v1/Figure 3C. COX IV.tif]

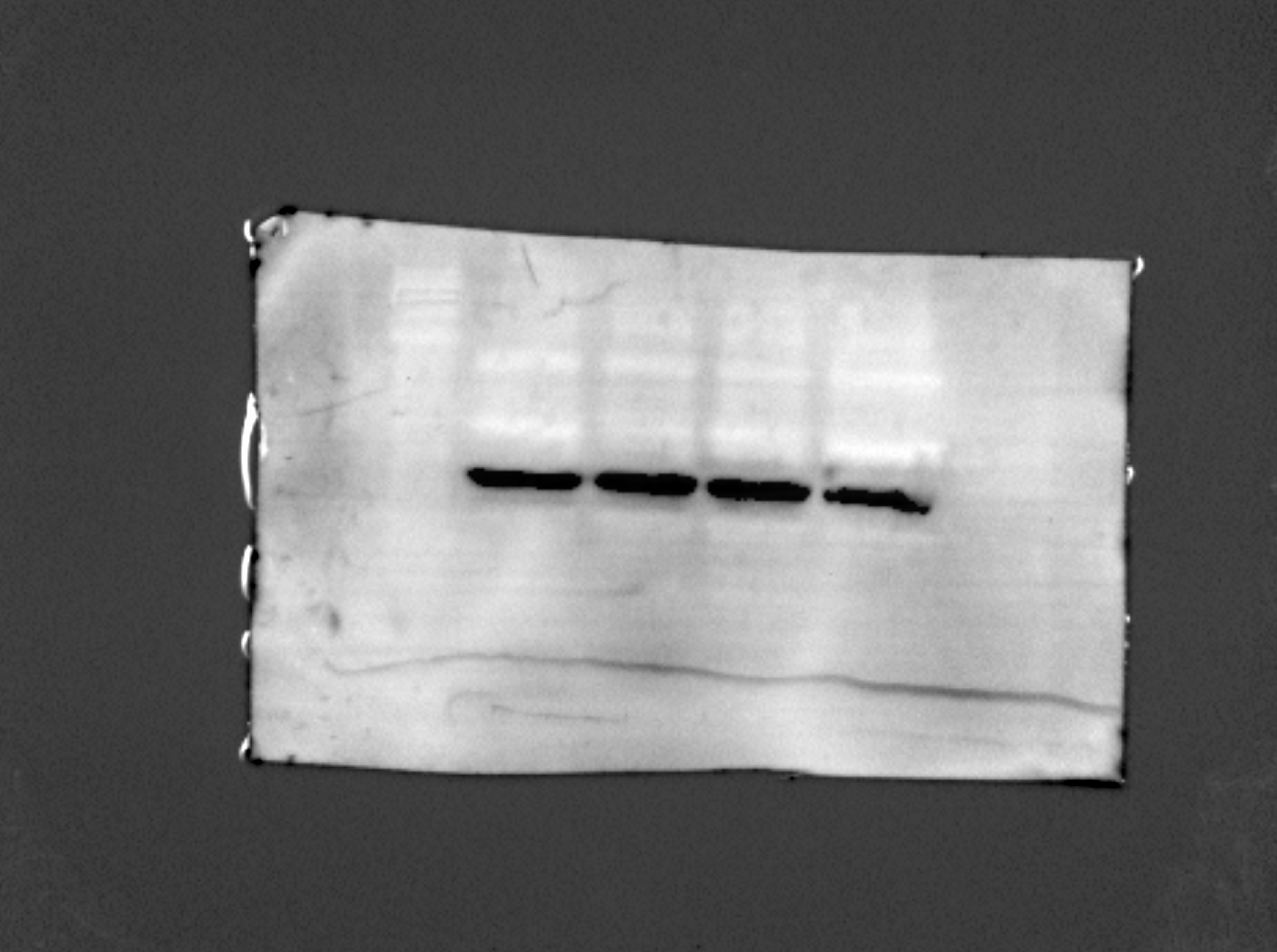

Supplement: Supplementary file 1 [file Datasheet1.zip › Data Sheet 1_v1/Figure 3C. GAPDH.tif]

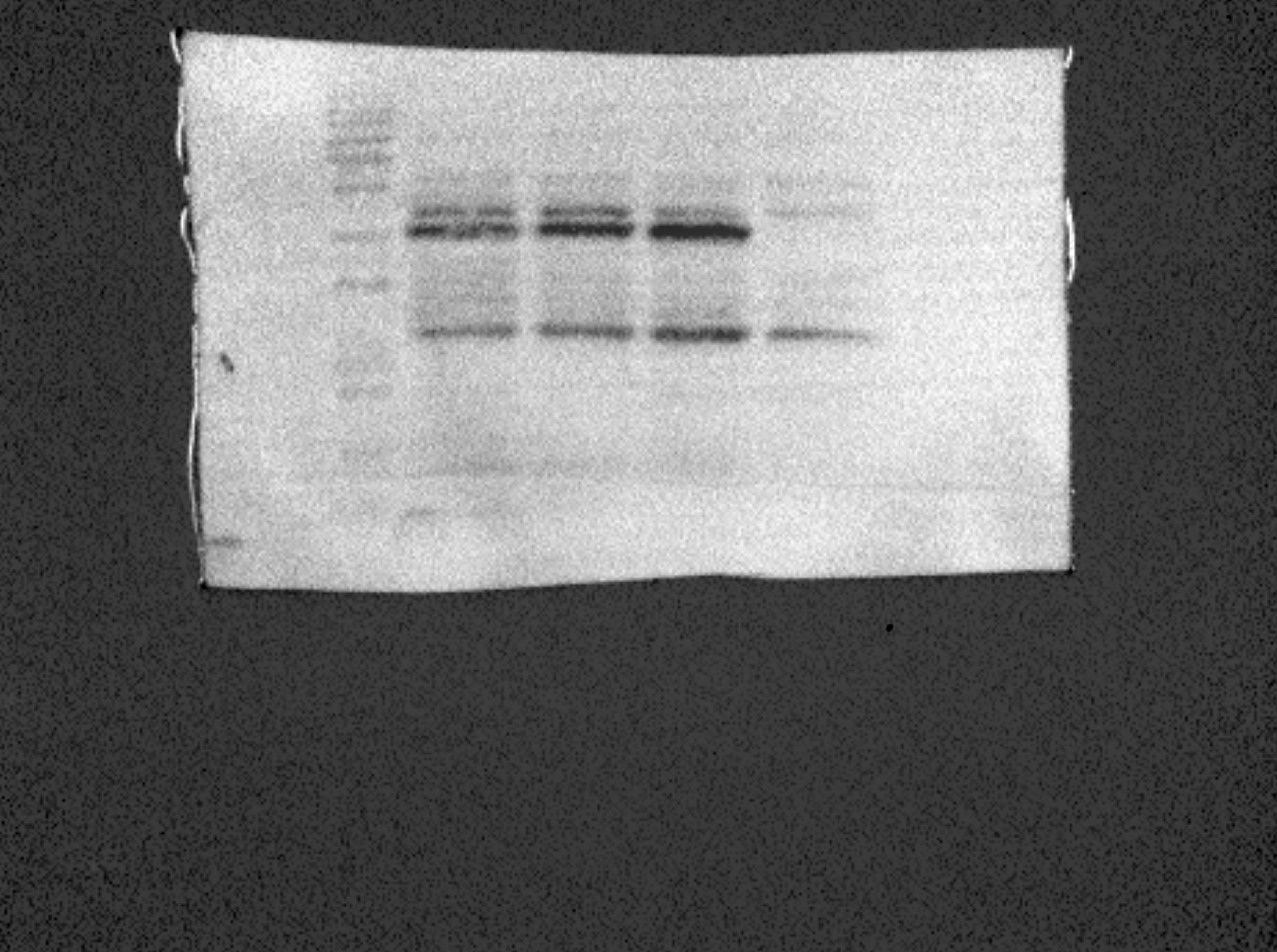

Supplement: Supplementary file 1 [file Datasheet1.zip › Data Sheet 1_v1/Figure 3C. TRNT1.tif]
