# Supplementary material for: Case report: Muscle involvement in a Chinese patient with TRNT1-related disorder
Source: Front Pediatr. 2023 May 5;11:1160107. doi: 10.3389/fped.2023.1160107 (PMC10196124; doi:10.3389/fped.2023.1160107)
Supplement: Supplementary file 3 [file Datasheet3.docx]

**Supplementary material**

**Trio-based whole-exome sequencing (****Trio-WES)**

Genomic DNA samples were extracted from the peripheral blood samples of the patient and his parents. Protein-coding exome enrichment was performed using the xGen Exome Research Panel v2.0 (IDT, Iowa, USA), which consists of 429,826 individually synthesized and quality-controlled probes. It targets a 39 Mb protein-coding region (19,396 genes) of the human genome and covers 51 Mb of end-to-end tiled probe space. High-throughput sequencing was performed by MGI DNBSEQ-T7 sequencing instruments (PE150). The paired-end reads were performed using Burrows-Wheeler Aligner (BWA) to the Ensemble GRCh37/hg19 reference genome.

**Trio-based whole-genome sequencing (Trio-WGS)**

Trio-WGS were used to further exclude other variants associated with the phenotype of the patient. The whole genomic library was constructed. High-throughput sequencing was performed on DNBSEQ-T7 series sequencer. Paired-end sequences were aligned to the GRCh37/hg19 reference genome using BWA.

**Single nucleotide polymorphism (SNP) array**

Infinium Global Screening Array (Illumina, San Diego, USA) was used to detect chromosome abnormalities including heteroploidy, deletion, duplication, and uniparental disomy.

**Mitochondrial DNA testing**

Full length mitochondrial DNA was amplified. DNA library was constructed by fragmenting the amplified product. Sequencing was performed by NextSeq500 (Illumina, San Diego, USA). The sequencing data were compared to the mitochondrial genome reference sequence NC_012920.1 (NCBI database) using NextGene V2.3.4 software.

**Western blot**

Total protein was extracted from the patient's muscle biopsy tissue. After SDS-PAGE, proteins were transferred to PVDF membranes (Millipore, Billerica, USA). The primary antibodies are anti-TRNT1 polyclonal antibody (ab224536, Abcam, USA), anti-COX IV monoclonal antibody (P01L08, Gene-Protein Link, China), and anti-GAPDH monoclonal antibody (#2118, Cell Signaling Technology, USA).
